# The Institutional Treatment of Consumption

**Published:** 1916-07-29

**Authors:** 


### THE INSTITUTIONAL TREATMENT OF CONSUMPTION.

### VII.—Notes on Administration in a Sanatorium.*

The administration of a modern sanatorium is so complex a business that a successful medical superintendent must be a man of high capabilities. To be a first-class clinician is not enough in these days of graduated labour; nor is it necessarily any very great advantage for the medical superintendent to have been a victim of pulmonary tuberculosis, as was once upon a time thought to be the case. Not only must the superintendent be able effectively to control a large staff and a number of often "difficult" convalescents, but he must possess a knowledge of many matters outside the realm of medicine, ranging from engineering to farming. It is essential for the success of a sanatorium that the medical chief should enjoy the confidence of the committee of management, or the local authority, and that he should be given a free hand. There must be no division of authority; the matron, the steward, and the engineer should be directly responsible to the medical superintendent, though they each would be in effective control of their respective subordinates.

Small sanatoria cannot be managed so economically as large ones; for one thing, the smaller ones cannot afford to offer sufficiently high salaries to attract a first-class man as a superintendent. A good man as chief is an invaluable asset to the committee of a sanatorium. He must know, for instance, enough about meat inspection and contracting to supervise the steward; enough about vegetable-growing or fruit-farming to know if the gardener is working to the advantage of the institution. His relations with the head-gardener are very important, on account of the necessity for strict medical supervision of the work done by patients in this department. Gardeners cannot be expected fully to appreciate the subtilties of auto-inoculation, and given a batch of patients their chief endeavour will be to get a certain amount of work done, instead of adapting the prescribed amount of exercise in each individual case to the horticultural needs of the moment. Hence the paramount importance of having all outdoor work overlooked by the medical superintendent or his assistant. The patients themselves require a watchful eye lest they should exceed the tasks allotted to each one. A system of coloured badges is a useful accessory; a certain colour represents a particular grade of exercise or labour, and the wearing of these badges indicates at once to the overseer the stage to which the patient has attained.

### Importance of Discipline.

It is hardly necessary to insist on the need for a strict discipline, especially among male patients. To deal justly, yet firmly, with the little acts of insubordination and the small mutinies which crop up from time to time, requires the exercise of great judgment and tact. Smoking in forbidden hours, and breaking bounds, are examples of the chief rule-breaking acts. Where men and women are accommodated in the same grounds, another source of difficulty arises, and constant surveillance is necessary to prevent too close acquaintance between the sexes. It may here be mentioned that, as a rule, male patients will outnumber the females, and the accommodation must be arranged accordingly. The difference in numbers is perhaps not so great now as it used to be, but there is undoubtedly more reluctance still on the part of women to report themselves and to submit to a course of treatment in an institution than there is among men of the working class; and it is also more difficult to persuade women to remain in a sanatorium for a prolonged period.

The number of nurses required in a sanatorium will be less in proportion to the number of patients than in a general hospital or in the hospital wards of a tuberculosis institution, for a great many patients will be able to make their own beds and will not require waiting upon for their meals. Probably from seven to ten nurses would be sufficient for a hundred average sanatorium patients. Ward-maids also may be limited, for, as has been indicated, a good deal of ward cleaning, etc., is done by patients, especially on the female side.

### The Kitchen and the Laundry.

In one direction at least in connection with the domestic staff it will pay to offer what may seem extravagant wages—that is, the head of the kitchen. This servant must not only be a good cook, but she must be an efficient manager. She should be encouraged to exercise some personal judgment and discrimination and not be tied down too closely to routine. Her opportunities for wasting good meat, etc., are large, and her capabilities of saving and yet providing agreeable and wholesome food should be encouraged freely. A salary of £60 or £80 per annum should be offered to a really competent woman, and it is well to arrange that the matron has not too much power of interference. In a well-organised institution the matron has quite enough work to occupy her without making her responsible for the culinary department.

It will be necessary to install a laundry in a detached building. The usual machinery for a sanatorium of 100 beds will include two washing machines, a centrifugal water-extracting machine, wash troughs, soap boiler, and starching trough. Six draw-out racks in the drying room heated by a current of hot air, an airing room adjoining, a large ironing machine for sheets, and a delivery and a receiving room well supplied with racks are some of the important items.

In dealing with patients who are nearly all able to move about it is a convenience to have a small room available in which the physician can make his examinations of the chest under more favourable conditions than in the ward or than in a sister's sitting-room, which might be within ear-

* The previous articles appeared on May 6 and 20, June 3 and 17, and July 1 and 15, 1916.

shot of the patient's resting place. Such a room might be provided in close proximity to the $x$-ray room and should adjoin a small ante-room for dressing and undressing.

While every sanatorium has a laboratory in which the examination of sputa, urine, blood, etc., is carried out, and where tuberculin is diluted under aseptic precautions ready for injection, not every institution has yet an $x$-ray room. It is true that in the majority of cases the patients enter the sanatorium already diagnosed, and that those sent for observation may be ultimately diagnosed by certain tests or by the subsequent course of the symptoms. Nevertheless the provision of an $x$-ray equipment in every sanatorium cannot be described as an unnecessary luxury. It is, in fact, an accessory of great assistance to the medical staff, not only in diagnosis or as a check on the physical sight, but in the treatment of pleurisies for instance. Now that the operation for the induction of an artificial pneumothorax is being more often performed, it cannot be too strongly insisted upon that where medical officers are expected to do this operation there must always be available an efficient $x$-ray apparatus. Those who are experienced in the technique of this often not sufficiently seriously regarded operation will bear out this statement, and also approve of another neglected precaution—namely, that the room in which the operation is done should be well warmed, if not positively hot, in order to avoid the shock of injecting cold nitrogen gas into the chest.

